# Craniomandibular Disorders in Pregnant Women: An Epidemiological Survey

**DOI:** 10.3390/jfmk5020036

**Published:** 2020-06-04

**Authors:** Grazia Fichera, Alessandro Polizzi, Simone Scapellato, Giuseppe Palazzo, Francesco Indelicato

**Affiliations:** 1 Department of General Surgery and Surgical-Medical Specialties, University of Catania, 95124 Catania, Italy; graziafichera@hotmail.it (G.F.); simonescapellato@hotmail.com (S.S.); gpalazzo@unict.it (G.P.); indelicato@policlinico.unict.it (F.I.); 2 Department of Biomedical, Odontostomatological Sciences and of Morphological and Functional Images, University of Messina, 98125 Messina, Italy

**Keywords:** temporomandibular joint, pregnancy, temporomandibular disorder, growth, clinical trial

## Abstract

Temporomandibular joint (TMJ) disorder has been reported to be 1.5 to two times more common in women than men. Such a gender-based difference could be attributed to behavioral, hormonal, anatomical, and psychological characteristics. Physiological hormonal differences between genders could be one of the possible explanations for the higher incidence of temporomandibular disorder (TMD) in women. As the plasma level of certain female hormones increases during gestation, it could be assumed that there is a higher prevalence of dysfunctional signs and symptoms in pregnant women. We performed an epidemiological survey based on screening for TMD in a group of 108 pregnant women and found that 72% of young women reported significant signs of TMJ disorders, 9% of the young women reported mild signs of TMJ disorders, and 19% of the included subjects reported no signs or symptoms of TMD. The presence of estrogen receptors in the temporomandibular joint of female baboons could be the basis of an explanation for the increased prevalence of dysfunction in young women reported in the literature and the high feedback we have seen of joint noises in pregnant women. On the basis of the present findings, it could be assumed that gestation period could represent a risk factor for craniomandibular dysfunctions.

## 1. Introduction

Dysfunction of the masticatory system, including the temporomandibular joint (TMJ), muscular and dental system, and the supporting bones, is called temporomandibular disorder (TMD) [1]. 

Adults are more affected by TMD as compared with children in a range of between 40% and 70%, however, a relevant high incidence of TMD has been found among subjects in mixed dental dentition [1,2,3]. Ethnicity, age, geographical location, and time of assessment influence the prevalence, causes, and factors that affect TMD, as well as the signs and clinical symptoms [4,5,6,7]. According to previous findings, TMD is generally reported to be 1.5 to two times more common in women as compared with men, and this difference is attributed to behavioral, hormonal, anatomical, and psychological factors [8,9]. In general, women are more affected than men by craniomandibular dysfunctions with a ratio of 4 to 1 [10,11,12,13,14]. The prevalence of clicks, headaches, teeth tightening, hypomobility, difficulty in chewing, and neuromuscular symptoms has been shown to be significantly higher among young women (<30 years) [15,16,17,18,19,20]. Moreover, there was a significant correlation between the severity of symptoms and age among women, and a relative reduction in clinical symptoms with age in both sexes [10,11,12,13,15,16,21,22]. Previous evidence reported that estrogen receptors are localized in the TMJ tissues, such as chondroid tissue of condyle and retrodiscal tissues [6,17,18,23,24,25,26,27]. In this respect, the hormone, estrogen, could influence the incidence of TMD, and its levels can affect the development, restitution, and metabolism of the temporomandibular joint, bone, and associated structures [7,23,25,28,29,30,31,32,33,34]. Estrogen can be related to TMD by regulating the pain mechanisms in the expressions of TMD [35]. For example, receptors for estrogens were found in both the peripheral and central nervous systems which would suggest that estrogens are capable of modifying pain signaling [36]. Moreover, estrogen receptors (ERα, ERβ) were also reported in the dorsal root ganglion (DRG) and in the trigeminal nerve nucleus. Estrogen can act as a pro- and anti-nociceptive, depending on the pain signaling type. In physiological pain, estrogen decreases pain, whereas, in inflammatory pain, the effect of estrogen depends on the inflammation type. In acute inflammatory pain, estrogen has an anti-nociceptive effect. On the contrary, in chronic inflammatory pain, this hormone has been documented to have a pro-nociceptive effect due to the presence of its receptors in tissues of both the peripheral and central nervous systems [36,37,38,39].

Thus, physiological hormonal differences between males and females could be one of the possible explanations for the higher incidence of TMD in the female. Given that the plasma level of some female hormones increases during gestation, it could be postulated that there is a higher prevalence of dysfunctional signs and symptoms in pregnant women than the frequencies reported in the literature for the same sex. In this respect, the aim of the present study was to assess the presence of TMJ disorders in a cohort of pregnant women.

## 2. Materials and Methods

### 2.1. Study Design

**Figure 1:**
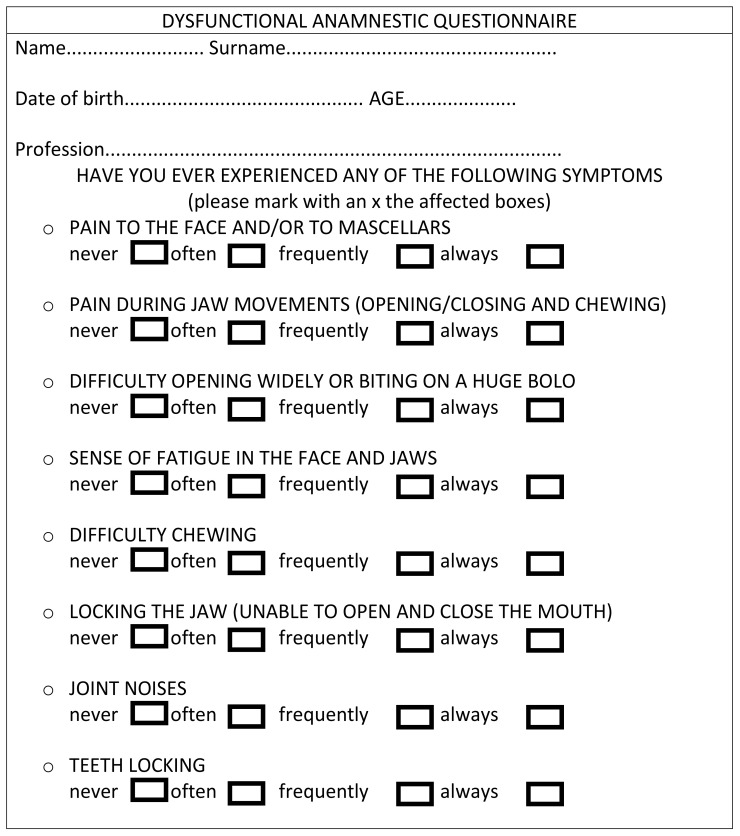
Example of the anamnestic Helkimo index.

This study followed the Helsinki Declaration on medical protocols and ethics and received positive response by the Approval Board of the School of Dentistry, University of Catania (protocol no. 14/19). The study sample included subjects followed at a private practice specialized in gynecology and gynecologic radiology, in Catania (Italy), between January 2016 and March 2020. For the investigation, subjects were recruited base on the following inclusion criteria: subjects at the sixth month of pregnancy, aged between 19 and 35 years, and absence of severe dental pathology potentially affecting the perception of TMD and craniofacial dysmorphism. The anamnestic data were collected using yes-no questionnaires in compliance with the Helkimo anamnestic dysfunction index (Ai) (Figure 1) and subjects were classified as Ai 0 (no symptoms), I (mild symptoms), and II (severe symptoms) based on the information obtained.

**Figure 2:**
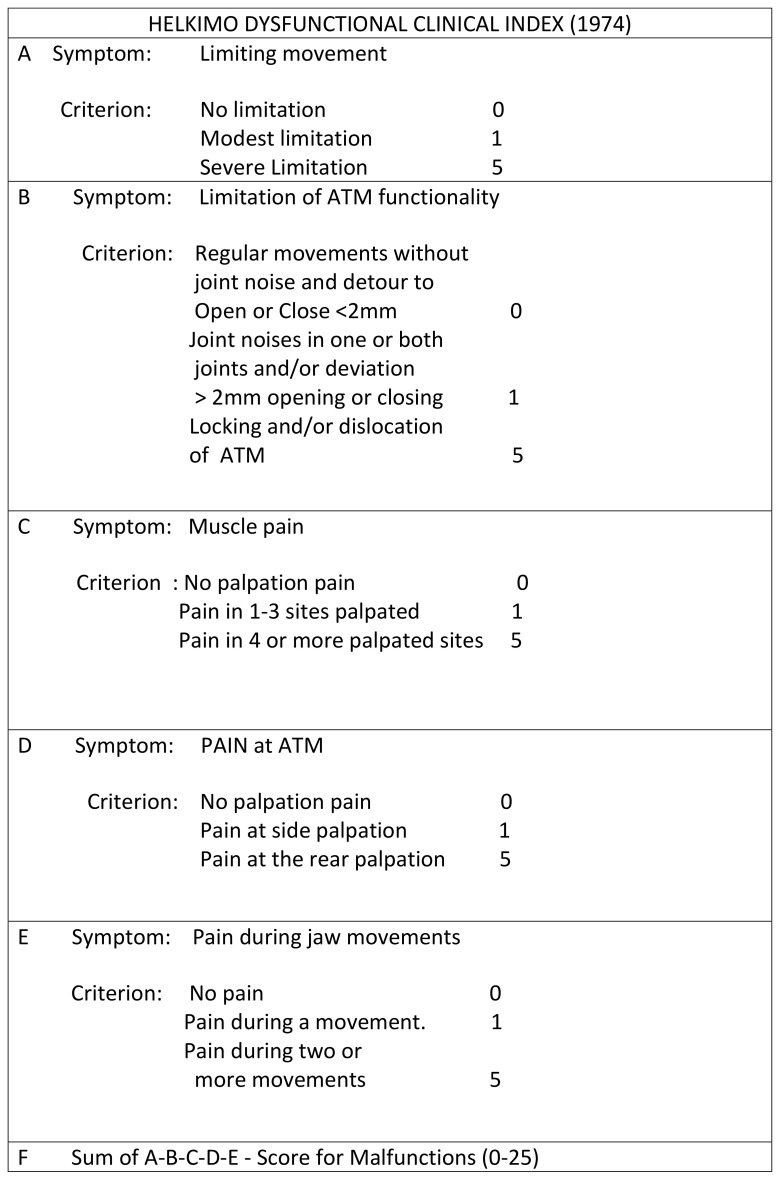
Example of the Helkimo clinical dysfunction index questionnaire filled out by one included patient.

Screening for craniomandibular dysfunctions was done by following the Helkimo dysfunction index guidelines (Figure 2) [40,41,42].

All examinations were performed by the same expert, blinded gnathologist. In particular, clinical examination of masticatory apparatus was performed using the Helkimo clinical dysfunction index (Di), which is based on five domains each evaluating one of the following signs of TMJ dysfunction: limited TMJ mobility, limited TMJ function, jaw muscle pain to palpation, TMJ pain to palpation, and pain during mandibular movement. Jaw movements were evaluated in order to highlight the presence of any limitations. The temporomandibular joint was examined for the diagnosis of joint noise, taking into account possible deviations and deflections of the lower median line over three chewing cycles. Palpation of the chewing muscles and the temporomandibular joint was carried out, and the mandible excursions were examined to assess the presence of pain. Scores for each of the domains were based on the three-level scale of severity, i.e., 0 (no symptoms), 1 (mild symptoms), and 5 (acute symptoms) and were summed up to obtain a total dysfunction score ranging from 0 to 25 points, with a high score indicating a higher temporomandibular dysfunction. In order to obtain comparative assessment, data of the study group were matched with a control sample of female subjects living in the same geographic area who had been referred to the same gynecology clinic for routine controls. The recruitment of the control group was based on the same inclusion criteria as the study group except for the pregnancy status.

### 2.2. Power Sample Analysis

A preliminary evaluation of sample size power was performed on 20 subjects (10 in the study group and 10 in the control group), the analysis suggested that 79 patients for each group were required to reach the 80% power to detect a mean difference of 7.2% [43] of incidence of clinically assessed joint click between study and control groups, with a confidence level of 95% and a beta error level of 20%. However, according to the inclusion criteria, it included 108 subjects in the study group and 90 subjects in the control group which increased the robustness of the data.

### 2.3. Statistical Analysis

The Kolmogorov–Smirnov test was preliminarily performed to test the normality of the data. Since the data showed homogeneous variance, parametric tests were used to evaluate and compare measurements.

For those subjects reporting positive values from the anamnestic index, data obtained from the dysfunction index were recorded on a specific spreadsheet. Datasets were analyzed using SPSS^®^ version 24 Statistics software (IBM Corporation, 1 New Orchard Road, Armonk, New York, USA). The incidence of TMD was detected as percentage values within the sample size. Chi-square test coefficient was used to compare the distribution data obtained from the anamnestic and dysfunction forms between each group. The level of significance was set at *p* < 0.05.

## 3. Results

One hundred and eight pregnant females (mean age 27.4 ± 3.8) were finally recruited to participate in the survey of the present investigation and 72% of young women reported significant signs of TMJ disorders. Thus, they were allocated to belong to the DII group (moderate dysfunctional) according to the anamnestic Helkimo index; 9% of young women reported slight signs of TMJ disorders and were allocated to the DI group (slightly dysfunctional). Consequently, 19% of the included subjects did not report signs or symptoms of TMD and were allocated to the the D0 group.

Ninety female subjects were enrolled in the control group (mean age 26.2 ± 4.5) and 41% of young women reported significant signs of TMJ disorders. Thus, they were allocated to the DII group (moderate dysfunctional) according to the anamnestic Helkimo index; 11% of young women reported slight signs of TMJ disorders and were allocated to the DI group (slightly dysfunctional). Consequently, 48% of the included subjects did not report signs or symptoms of TMD and were allocated to the D0 group.

Table 1 shows the distribution of data obtained with the anamnestic Helkimo index and assessed using the Chi-square test (*p* < 0.05). Table 2 shows the distribution of data obtained with the Helkimo dysfunction index and assessed using the Chi-square test. In both groups, all subjects in the DII group reported TMJ click, whereas subjects in the DI group reported muscular pain (*p* < 0.05).

## 4. Discussion

From the literature review, it can be seen that the incidence of craniomandibular dysfunctions, except for a few exceptions, shows no gender differences in school-age patients, whereas, for adults, prevalence of signs is observed dysfunctional in the female sex. It can be hypothesized with great certainty that, after adolescence, physiological, psychological, and morphological differences are taking over. With regard to physiological differences, in addition to the effects of hormonal regulation of metabolic activities, women are much more susceptible to pulp stimulation pain. Men tolerate deep pain much more readily than women, and pain tolerance in general and the threshold of skin pain are lower in women than in men [44,45,46].

Patients with craniomandibular dysfunction, and especially women, similar to periodontitis [28,47,48,49,50,51,52,53] have a lower pain tolerance than the control group [42,54], and a lower pain threshold [55,56]. In addition, emotional stress increases pain by accelerating activities in the neural system, which is, in turn, stimulated by harmful impulses. Anxiety, depression, anger, induce autonomous, visceral and skeletal activities, and the interactions between these biological systems are illustrated in the pain-anxiety-tension cycle proposed by some authors to explain some forms of acute pain, and often observed in pathologies affecting the skeletal-muscular system [57,58,59,60,61].

Pain causes anxiety, which induces both prolonged muscle spasms at pain sites and trigger points, as well as vasoconstriction, ischemia, and release of pain-mediating substances. Similarly, depression has a profound effect on peripheral symptoms, interacting through the thalamus and hypothalamus. Catecolo-methyltransferases (COMT) is an important enzyme that inactivates neurotransmitters and regulators of the central and peripheral nervous system. Patients with craniomandibular dysfunction have a low erythrocyte level of COMT as compared with control subjects, and therefore appear to be predisposed to depression. With a few exceptions, women suffer much more than men from depression and this psychophysiological difference could explain the results of his studies [62].

Depression is easier to match among young women and usually decreases with age; however, symptoms of depression are less common among young males [24]. This could justify the significant correlation between the severity of symptoms and age for women, and the decrease in the number of symptoms for both sexes previously found [21,63,64]. The authors also saw that young women have a high incidence of clicks, headaches, teeth tightening, hypomobility, difficulty in chewing, and neuromuscular symptoms. This could be explained by the fact that women are much more susceptible to tissue alterations and, in particular, to those of the condyle-disc complex [24,64].

The teeth tightening found in young women would represent a functional expression of specificity of response originating from the activity of large muscles and high residual muscle tension. Chronic muscle hyperactivity with the maintenance of high residual muscle tension could initiate numerous pathophysiological mechanisms [49,65,66,67].

It must also be said that women from puberty to menopause have a hormonal structure that varies cyclically in a physiological way. During pregnancy, this situation changes further, as, after fertilization, the lute body remains active, magnifying large amounts of estrogen and progesterone. After the third month, it is no longer essential for the continuation of pregnancy, as its endocrine function changes to the placenta, so it regresses. The placenta, as well as an organ responsible for the exchanges between the fetus and the mother, is also an endocrine organ, which separates at least four types of hormones, i.e., a gonadotropin, estrogen, progesterone, and relaxin [67]. The effects of sex hormones in target tissues are varied and very significant. They stimulate collagen and protein synthesis, increased vasal reactivity, alteration of endothelial permeability, and prostaglandins synthesis [67,68,69,70]. They also have an effect on the immune system, having a role in antibody formation and in stem cell differentiation and proliferation. They inhibit leucocyte proliferation in rats and, in humans, suppress the transformation of lymphocytes. Estrogens also tend to increase the immune response, as opposed to androgens [71].

The presence of estrogen receptors in the temporomandibular joint of female baboons [16,71] could be the basis of an explanation for the higher prevalence of the dysfunction in young women reported in the literature [72,73,74,75,76,77,78,79,80,81,82,83], and the high feedback we have noted of joint noises in pregnant women.

Relaxin, which is mainly found in serum and mammalian tissues, especially during pregnancy, has a series of actions that together promote gestation and prepare the female reproductive apparatus for childbirth. The connective tissue of pubic symphyses causes the transformation from cartilage to the more fluid and flexible with ligament formation between the two pubic bones. They increase collagen texture and extensibility [83,84,85] and dilate and makes the cervix softer.

On the basis of previous evidence confirming the prevalence of TMD in female [24,26,72], our findings would suggest that pregnancy can be more susceptible from TMD and that gestation is a predisposing factor for craniomandibular dysfunctions [58,59,60,61,62,63].

The hypothesis that relaxin, reaching high levels during pregnancy, affects the tenderness of joint tissues, including the TMJ [74,75], could explain the high incidence of clicks in the pregnant young women found in the present study. Further studies, based on this assumption, are warmly required in order to provide new information on the potential effects of hormone levels on the development of TMD.

The limitation of this study is the absence of a quantitative distinction between physical assessment and psychological assessment of TMD and further studies should include this comparative evaluation in the methodology.

## 5. Conclusions

Female subjects in pregnancy status could be more susceptible to TMD due to a physiological increment of estrogenic hormones levels. However, further studies are needed to better understand the role of TMD during pregnancy.

## Figures and Tables

**Table 1:** Data distribution from the anamnestic form (Helkimo’s index). Significance set at *p* < 0.05 according to the Chi-Square test.

		TMJ Disfunction			
		Positive	Negative	Total	
Study Group	Observed	87	21	108	*p* < 0.05
	Percentage (%)	81	19	100	
Study Group	Observed	47	43	90	
	Percentage (%)	52	48	100	

**Table 2:** Data distribution from the anamnestic form (Helkimo’s index). Significance set at *p* < 0.05 according to the Chi-Square test.

		Articular Signs	Muscular Pain	Total	
Study Group	Observed	87	0	87	*p* < 0.05
	Percentage (%)	100	0	100	
Study Group	Observed	37	10	47	
	Percentage (%)	79	21	100	
